# Individual, facility, and program factors affecting retention in a national weight management program

**DOI:** 10.1186/1471-2458-14-363

**Published:** 2014-04-15

**Authors:** Bonnie Spring, Min-Woong Sohn, Sara M Locatelli, Sattar Hadi, Leila Kahwati, Frances M Weaver

**Affiliations:** 1 Department of Preventive Medicine, Feinberg School of Medicine, Northwestern University, Chicago, IL, USA; 2 Center of Innovation for Complex Chronic Healthcare, Hines VA Hospital, Hines, IL, USA; 3 Center for Healthcare Studies, Feinberg School of Medicine, Northwestern University, Chicago, IL, USA; 4 Patient Care Services, Hines VA Hospital, Hines, IL, USA; 5 National Center for Health Promotion and Disease Prevention, Office of Patient Care Services, Veterans Health Administration, US Department of Veterans Affairs, Durham, NC, USA; 6 Health Services Research Program, Stritch School of Medicine, Loyola University Chicago, Maywood, IL, USA

**Keywords:** Overweight, Obesity, Veterans, Veterans health, Health education, Weight loss

## Abstract

**Background:**

High attrition is a common problem for weight loss programs and directly affects program effectiveness. Since 2006, the Veterans Health Administration (VHA) has offered obesity treatment to its beneficiaries through the MOVE! Weight Management Program for Veterans (MOVE!). An early evaluation of this program showed that attrition rate was high. The present study examines how individual, facility, and program factors relate to retention for participants in the on-site MOVE! group program.

**Methods:**

Data for all visits to MOVE! group treatment sessions were extracted from the VHA outpatient database. Participants were classified into three groups by their frequency of visits to the group program during a six month period after enrollment: early dropouts (1 – 3 visits), late dropouts (4 – 5 visits), and completers (6 or more visits). A generalized ordered logit model was used to examine individual, facility, and program factors associated with retention.

**Results:**

More than 60% of participants were early dropouts and 11% were late dropouts. Factors associated with retention were older age, presence of one or more comorbidities, higher body mass index at baseline, lack of co-payment requirement, geographic proximity to VA facility, addition of individual consultation to group treatment, greater program staffing, and regular, on-site physical activity programming. A non-completion rate of 74% for on-site group obesity treatment poses a major challenge to reducing the population prevalence of obesity within the VHA.

**Conclusions:**

Greater attention to individualized consultation, accessibility to the program, and facility factors including staffing and physical activity resources may improve retention.

## Background

High attrition is a common problem for weight-loss programs [1,2] and extensive research has demonstrated that participation in a greater number of treatment sessions predicts better weight loss outcomes [3-6]. However, there is limited information on factors associated with retention in on-site weight-loss programs. Especially lacking is information on program and facility characteristics that affect retention.

The Veterans Health Administration’s (VHA) nationwide MOVE! Weight Management Program represents a model system for population-level obesity management. General program guidelines align with the U.S. Preventive Services Task Force’s (USPSTF) directives which provide a Class B recommendation (fair evidence of benefit) for intensive or recurrent behavioral intervention for all obese adults [7]. USPSTF defines intensive treatment as more than one contact per month for at least the first three months [7]. Analysis of 2007 VHA MOVE! data indicated that fewer than 18% of participants had attended at least six treatment sessions in the six months after initiating treatment [8]. In comparison, attrition in commercial weight loss programs approaches 56% by six months after enrollment [1,9]. This suggests that MOVE! may have a more serious problem with retaining patients than many commercial programs. In this study, national MOVE! data were used to identify individual, facility, and program factors that predict retention in the MOVE! program.

## Methods

### Program and context

The MOVE! program was developed by the VHA to provide weight management services to its beneficiaries and was implemented nationally in 2006 [10]. The target population includes individuals who are obese (BMI ≥ 30 kg/m^2^) or overweight (BMI 25 – 30 kg/m^2^) if they have elevated waist circumference or with an obesity-related condition such as diabetes and hypertension. By 2008, nearly all VHA facilities (98.7%) offered MOVE! and more than 100,000 individuals had participated in MOVE! [10].

An early evaluation of MOVE! released by the national MOVE! program office suggests that the program has been successful in helping individuals to maintain or reduce their weight. About 70% of MOVE! participants with six or more visits to the program either stopped gaining weight or lost weight and 22% of men and 24% of women achieved 5% or greater weight loss after six months in the program [8,11].

Design of the MOVE! program was based on National Institutes of Health and VHA clinical practice guidelines [12,13]. It is offered as part of patients’ ongoing care and provided through primary care clinics, or via interdisciplinary programs that involve individual or group care. The program focuses on self-management regarding diet, physical activity, and behavior modification through individual or group treatments. Local programs can incorporate different approaches for weight reduction that include (a) self-management support through group sessions, individual consultation, or telehealth, (b) use of weight-loss medications, (c) short-term residential intensive treatment (e.g., in a domiciliary or hospital), and (d) bariatric surgery.

An in-person, on-site group program led by health professionals (e.g., dietitians, nurse professionals, or primary care physicians) is the most widely used modality. The group program includes up to 12 sessions, comprised of an orientation session and core sessions covering lectures and group discussions about nutrition, physical activity and behavior modification discussions [10,14]. Each VHA facility configures its group program to suit its own patient mix, facility, space, and staffing characteristics. For this reason, there is a wide variability in program implementation, effectiveness, and attrition rates from site to site. As an example, the VA Connecticut Healthcare System reports that it organized the MOVE! group program as one-hour weekly sessions that continue over 10 weeks and then cycle back to the beginning [15]. Eligible individuals can join the group sessions at any point and are encouraged to achieve their weight-loss goals by remaining in the program for as many sessions as they wish.

Individual consultation is available as part of MOVE! for persons who need individualized care due to specific disabilities or medical conditions. The specialist can be a dietitian, physical therapist, behavioral health professional, or medical specialist. The goal of individual consultation is to develop a treatment plan that is tailored to the particular patient’s needs.

### Design and data sources

VHA outpatient records were examined to identify the date on which an individual attended a MOVE! group session for the first time (index date). All individuals whose index date fell between October 1, 2007 and September 30, 2008 (fiscal year 2008; all years henceforth are fiscal years) were considered for entry into the study cohort (N = 29,979). Patient-level data were acquired through the VHA Medical SAS Outpatient Datasets, VHA Vital Status File, Assistant Deputy Under Secretary (ADUSH) Enrollment File, Corporate Data Warehouse (CDW) Vital Signs Dataset, and the US Department of Veterans Affairs (VA) Planning System Support Group. MOVE! program and facility characteristics were obtained through MOVE! Annual Report data.

**Figure 1:**
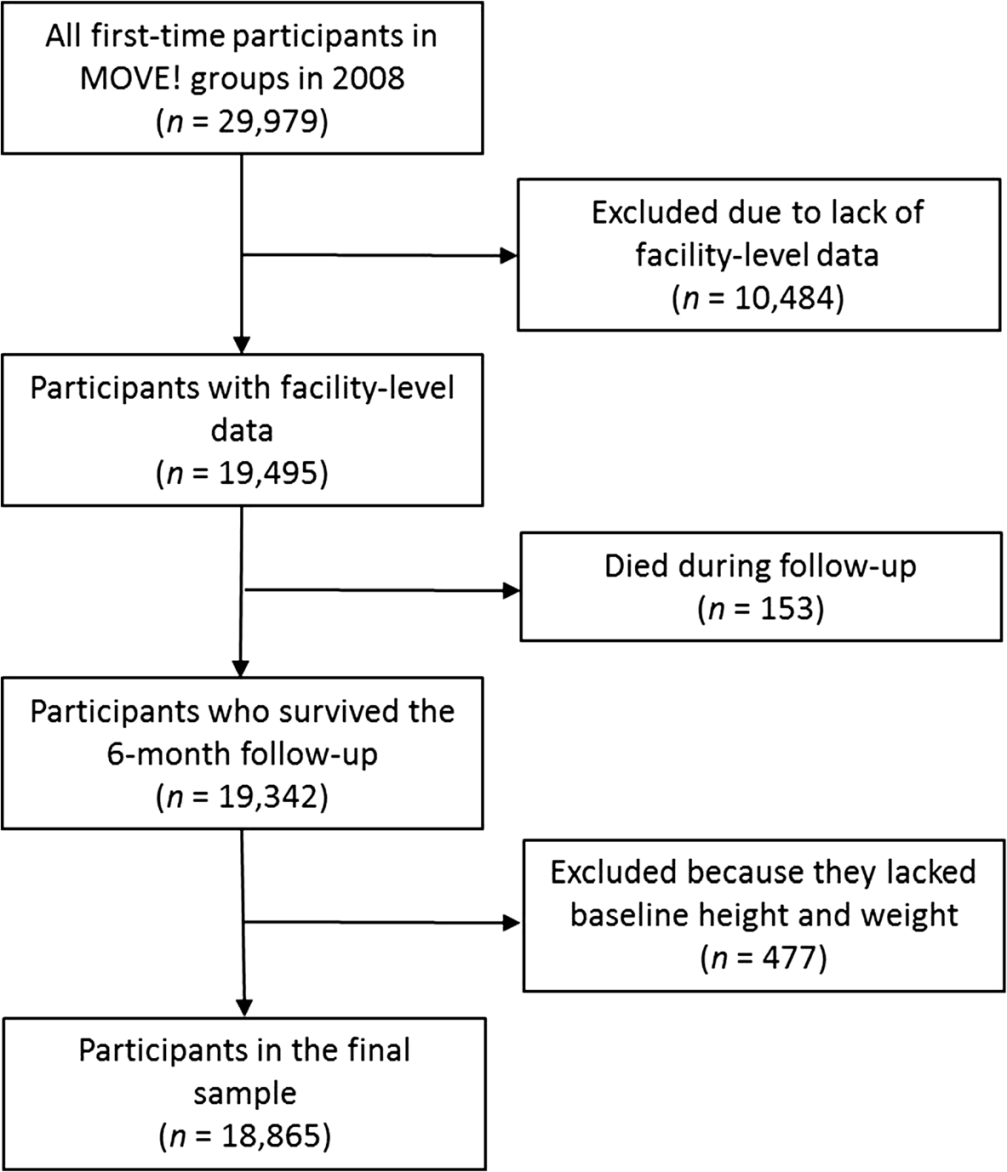
Study flow diagram.

Individuals who participated in MOVE! at clinics whose facility-level data were unavailable (n = 10,484), those who died before the end of follow-up (n = 153), or those who lacked a measure of height and weight during one year before the index date (n = 477) were excluded from analysis. The final cohort included 18,865 individuals accounting for 63% of all new MOVE! group participants in 2008. See the Study Flow Diagram in Figure 1.

### Measurement of treatment retention

First-time MOVE! participants in 2008 were followed for six months to count all visits to MOVE! group sessions during the six-month period from the index date. Because the national MOVE! Program Office within the VHA National Center for Health Promotion and Disease Prevention (NCP) recommends six or more visits over 6 months as a goal for an effective intervention [8], six visits were used as a threshold for meeting the overall or long-term retention goal. In addition, four visits were used as meeting the minimum or short-term retention goal. These thresholds were used to categorize individuals into three retention groups: early dropouts (1 – 3 visits), late dropouts (4 – 5 visits), and completers (≥6 visits).

### Patient characteristics

Individual demographic characteristics including age, sex, race/ethnicity, and marital status were obtained from administrative records. Body mass index (BMI) was computed from average height and weight during one year before the index date; participants were categorized as overweight (BMI = 25.0-29.9 kg/m^2^), obese (BMI = 30.0-39.9 kg/m^2^), or morbidly obese (BMI ≥ 40.0 kg/m^2^). The Elixhauser Comorbidity Index [16] was used to identify comorbidities during one year before the index date; we assigned patients to four groups based on their number of co-existing medical conditions (none, one, two, or ≥ three).

### Accessibility characteristics

Accessibility of MOVE! group sessions was measured by the VA enrollment priority and by geographic distance between a patient’s residence and the MOVE! clinic. Copayments for MOVE! visits were eliminated in June, 2008 [10]. However, patients may schedule MOVE! visits to coincide with other clinic visits for which copayments may be assessed. The VA enrollment priority was used to assign patients into three copayment groups: those without copayments for any VA care, those with copayments for some services, and those with copayments for all services they utilize. The geographic distance was computed using the computer algorithm provided elsewhere [17] based on patient home zipcodes and clinic locations.

### Program and facility characteristics

The use of individual consultation was identified at the patient level and categorized into three groups: never used consultation, used it on or before the index date, and used it after the index date.

Information about the MOVE! program at each facility was collected from the NCP Annual Report, which included MOVE! staffing levels, programs implemented (e.g., individual self-management support through visits or by phone, weight-loss medication, intensive residential treatment, and bariatric surgery), and weight loss strategies used as part of MOVE! at each facility. The staffing level was measured as the total number of MOVE!-specific full-time equivalents per 1,000 unique group participants at the facility. Also included was whether each of 10 common weight reduction strategies was used “often” or “almost always”: explicit goal setting, self-monitoring, behavior modification, relapse prevention (weight loss maintenance strategies), mindfulness-based approaches, cognitive therapies, use of social support/pressure, use of incentives/rewards, low-calorie diet plans, and regular on-site physical activity sessions.

### Statistical analysis

The focus of our analysis was to identify factors associated with short-term and long-term retention. Three retention groups were compared using bivariate analyses (Tables 1 and 2) and generalized ordered logistic regression analysis (Table 3). The regression model, adjusted for patient, accessibility, and program and facility characteristics, produces two sets of estimates. The first set compares early dropouts with a combined group comprised of late dropouts and completers. The second set compares early and late dropouts with completers. All estimates provide the likelihood of a patient being in the higher-ordered group compared with all patients in the lower groups combined [18]. All statistical analyses were conducted using SAS v9.2 and STATA SE 11.0.

**Table 1:** Distribution of MOVE! group participants by individual and accessibility characteristics and number of group sessions attended (N = 18,865)

**Characteristic**	**All, N (Col%)**	**Percent of persons in category**			**P-value**
		**< 4 Visits**	**4 - 5 Visits**	**6+ Visits**	
All	18,865 (100.0%)	63.8%	10.6%	25.6%	
Age					
< 55	6,989 (37.0%)	69.2%	10.8%	20.0%	< 0.001
55 – 64	8,039 (42.6%)	61.7%	10.6%	27.6%	
65 or older	3,837 (20.3%)	58.1%	10.3%	31.6%	
Sex					
Female	2,633 (14.0%)	61.8%	12.8%	25.4%	< 0.001
Male	16,232 (86.0%)	64.1%	10.3%	25.7%	
Race/Ethnicity*					
NH White	10,515 (55.7%)	60.0%	11.0%	28.9%	< 0.001
NH Black	4,189 (22.2%)	64.4%	13.0%	22.6%	
Hispanic	1,097 (5.8%)	63.9%	8.9%	27.2%	
Other/Unknown	3,064 (16.2%)	75.6%	6.5%	17.9%	
Marital Status					
Not Married	9,352 (49.6%)	64.2%	10.5%	25.3%	0.455
Married	9,513 (50.4%)	63.3%	10.7%	25.9%	
Comorbidities					
None	3,374 (17.9%)	70.0%	8.7%	21.3%	< 0.001
1	4,769 (25.3%)	64.2%	11.2%	24.6%	
2	4,734 (25.1%)	62.0%	10.9%	27.1%	
3 or more	5,988 (31.7%)	61.3%	11.0%	27.7%	
Body Mass Index (kg/m^2^)					
Overweight (25–29.9)	2,514 (13.3%)	68.8%	10.1%	21.1%	< 0.001
Obese (30–39.9)	11,397 (60.4%)	64.5%	10.5%	25.0%	
Morbidly obese (40 or over)	4,954 (26.3%)	59.6%	11.2%	29.2%	
Distance to Facility					
<10 miles	11,885 (63.0%)	60.4%	11.1%	28.5%	< 0.001
10 - 19.9 miles	3,739 (19.8%)	68.6%	9.6%	21.8%	
20 - 29.9 miles	1,704 (9.0%)	70.7%	9.6%	19.8%	
30 miles or more	1,537 (8.1%)	70.1%	10.6%	19.3%	
Copayment Status					
None	5,728 (30.4%)	61.2%	11.2%	27.6%	< 0.001
Some	10,204 (54.1%)	65.4%	10.4%	24.2%	
All	2,933 (15.5%)	63.1%	10.2%	26.7%	
Individual Consultation					
Never	9,764 (51.8%)	69.8%	9.6%	20.6%	< 0.001
On or Before	5,944 (31.5%)	54.5%	12.9%	32.6%	
After	3,157 (16.7%)	62.5%	9.6%	27.9%	

*NH = non-Hispanic.

**Table 2:** Distribution of MOVE! participants by facility and program characteristics and number of group sessions attended (N = 18,865)

**Variable**	**Clinics, N (Col%)**	**All enrollees, N (Col%)**	**< 4 Visits**	**4 - 5 Visits**	**6+ Visits**	**P-value**
All	132 (100.0%)	18,865 (100.0%)	64.1%	10.7%	25.0%	
**MOVE! Services Implemented**						
Self-Management Support	118 (85.8%)	16,191 (83.2%)	62.9%	10.8%	26.3%	< 0.001
Weight-loss Medication	73 (55.3%)	11,806 (62.6%)	62.7%	12.0%	25.4%	< 0.001
Intensive Medical Care	31 (23.5%)	4,909 (26.0%)	63.8%	15.0%	21.2%	< 0.001
Bariatric Surgery	36 (27.3%)	6,756 (35.8%)	64.6%	8.4%	27.0%	< 0.001
**Program Implementation**						
MOVE! Physician Champion	119 (90.2%)	17,515 (92.8%)	64.5%	10.8%	24.8%	0.14
MOVE! IT Support	111 (84.1%)	16,692 (88.5%)	63.4%	11.4%	25.3%	< 0.001
Total FTEs/1,000 Participants*						
Low (< 3.35)	24 (18.2%)	6,310 (33.4%)	72.0%	10.0%	18.0%	< 0.001
Medium (3.35 – 7.60)	29 (22.0%)	6,327 (33.5%)	64.7%	9.1%	26.3%	
High (7.61+)	79 (59.8%)	6,228 (33.0%)	55.9%	13.2%	30.9%	
**Program Strategies**^**†**^						
Explicit Goal Setting	126 (95.5%)	17,076 (90.5%)	63.2%	10.9%	25.9%	< 0.001
Self-Monitoring	120 (90.9%)	15,841 (84.0%)	62.1%	10.9%	27.0%	< 0.001
Behavior Modification	123 (93.2%)	16,941 (89.8%)	63.5%	10.4%	26.0%	< 0.001
Relapse Prevention	100 (75.8%)	13,757 (72.9%)	61.1%	10.2%	28.8%	< 0.001
Mindfulness-Based Approach	85 (64.4%)	11,936 (63.3%)	60.5%	9.9%	29.6%	< 0.001
Cognitive Therapies	90 (68.2%)	12,875 (68.2%)	61.1%	10.6%	28.3%	< 0.001
Social Support/Pressure	102 (77.3%)	14,944 (79.2%)	62.1%	10.1%	27.8%	< 0.001
Incentives/Rewards	28 (21.2%)	5,326 (28.2%)	59.1%	12.0%	28.8%	< 0.001
Low Calorie Diet Plans	41 (31.1%)	5,848 (31.0%)	58.9%	11.1%	30.0%	< 0.001
On-Site Physical Activity Sessions	38 (28.8%)	5,116 (27.1%)	54.5%	14.0%	31.6%	< 0.001

*FTE = full-time equivalent.

^**†**^Facilities can choose to use more than one strategy.

**Table 3:** Generalized ordered logit odds ratios and 95% confidence intervals for short-term and longer-term retention in the MOVE! group Program (N = 18,865)†

**Variable**	**Four or more visits (reference: < four Visits)**			**Six or more visits (reference: < six visits)**		
	**OR (95% CI)**		**P-value**	**OR (95% CI)**		**P-value**
**Individual Factors**						
Age [< 55]						
55 - 64	1.431	(1.317-1.554)	< 0.001	1.534	(1.401-1.680)	< 0.001
75+	1.581	(1.393-1.794)	< 0.001	1.827	(1.606-2.079)	< 0.001
Male [Female]	0.746	(0.663-0.840)	< 0.001	0.746	(0.663-0.840)	< 0.001
Race/Ethnicity [NH White]*						
NH Black	0.893	(0.766-1.041)	0.149	0.789	(0.673-0.925)	0.003
Hispanic	0.913	(0.726-1.148)	0.436	0.913	(0.726-1.148)	0.436
Other/Unknown	0.552	(0.479-0.635)	< 0.001	0.552	(0.479-0.635)	< 0.001
Married [Not Married]	1.017	(0.936-1.104)	0.695	1.017	(0.936-1.104)	0.695
Comorbidities [None]						
1	1.167	(1.046-1.301)	0.006	1.167	(1.046-1.301)	0.006
2	1.202	(1.092-1.323)	< 0.001	1.202	(1.092-1.323)	< 0.001
3 or more	1.174	(1.052-1.312)	0.004	1.174	(1.052-1.312)	0.004
Body Mass Index [25–29.9 kg/m2]						
30 - 39.9	1.221	(1.088-1.371)	0.001	1.221	(1.088-1.371)	0.001
40 or over	1.471	(1.280-1.691)	< 0.001	1.471	(1.280-1.691)	< 0.001
**Accessibility Factors**						
Distance to the MOVE Clinic [<10 miles]						
10 - 19.9 miles	0.751	(0.655-0.861)	< 0.001	0.751	(0.655-0.861)	< 0.001
20 - 29.9 miles	0.666	(0.538-0.823)	< 0.001	0.666	(0.538-0.823)	< 0.001
30 miles or more	0.681	(0.572-0.812)	< 0.001	0.681	(0.572-0.812)	< 0.001
Copayment Status [None]						
Some	0.848	(0.783-0.917)	< 0.001	0.848	(0.783-0.917)	< 0.001
All	0.858	(0.771-0.955)	0.005	0.858	(0.771-0.955)	0.005
**Facility Factors**						
Individual Consultation [Never]						
On or Before	1.684	(1.300-2.181)	< 0.001	1.684	(1.300-2.181)	< 0.001
After	1.497	(1.054-2.126)	0.024	1.719	(1.240-2.382)	0.001
Level of Implementation						
Self-Management	0.509	(0.337-0.768)	0.001	0.509	(0.337-0.768)	0.001
Weight-Loss Medication	0.976	(0.697-1.366)	0.886	0.852	(0.622-1.165)	0.315
Intensive Medical Care	0.980	(0.716-1.342)	0.902	0.749	(0.568-0.987)	0.040
Bariatric Surgery	1.046	(0.754-1.451)	0.789	1.351	(1.011-1.806)	0.042
Program Implementation						
Physician champion	0.965	(0.656-1.420)	0.856	0.965	(0.656-1.420)	0.856
IT Support	0.982	(0.653-1.476)	0.929	0.851	(0.594-1.221)	0.382
Staffing Level [Low]						
Medium	1.368	(0.921-2.034)	0.121	1.368	(0.921-2.034)	0.121
High	1.958	(1.326-2.890)	0.001	1.958	(1.326-2.890)	0.001
**Program Strategies**						
Explicit goal setting	2.387	(0.744-7.658)	0.143	2.387	(0.744-7.658)	0.143
Self-monitoring	1.511	(0.927-2.462)	0.097	1.511	(0.927-2.462)	0.097
Behavior modification	0.500	(0.189-1.320)	0.162	0.500	(0.189-1.320)	0.162
Relapse Prevention	0.710	(0.447-1.127)	0.146	0.918	(0.593-1.420)	0.701
Mindfulness-Based Approach	1.289	(0.849-1.957)	0.233	1.289	(0.849-1.957)	0.233
Cognitive Therapies	0.934	(0.588-1.484)	0.772	0.934	(0.588-1.484)	0.772
Social Support/Pressure	1.527	(0.797-2.925)	0.202	1.527	(0.797-2.925)	0.202
Incentives/Rewards	1.444	(1.014-2.057)	0.042	1.444	(1.014-2.057)	0.042
Low Calorie Diet Plans	0.908	(0.644-1.281)	0.583	0.908	(0.644-1.281)	0.583
On-Site Physical Activity Sessions	1.512	(1.061-2.155)	0.022	1.512	(1.061 -2.155)	0.022

†Referent category in brackets.

*NH = non-Hispanic.

### Approval for research involving human subjects

This study was approved by the Institutional Review Board at the Hines VA Hospital, Hines, Illinois, USA.

## Results

Of 18,865 patients in the study cohort, 80% were <65 years old. Females were disproportionately more likely to participate: they accounted for 14% of group participants (Table 1), though they comprised 5.5% of VHA users [19,20].

**Figure 2:**
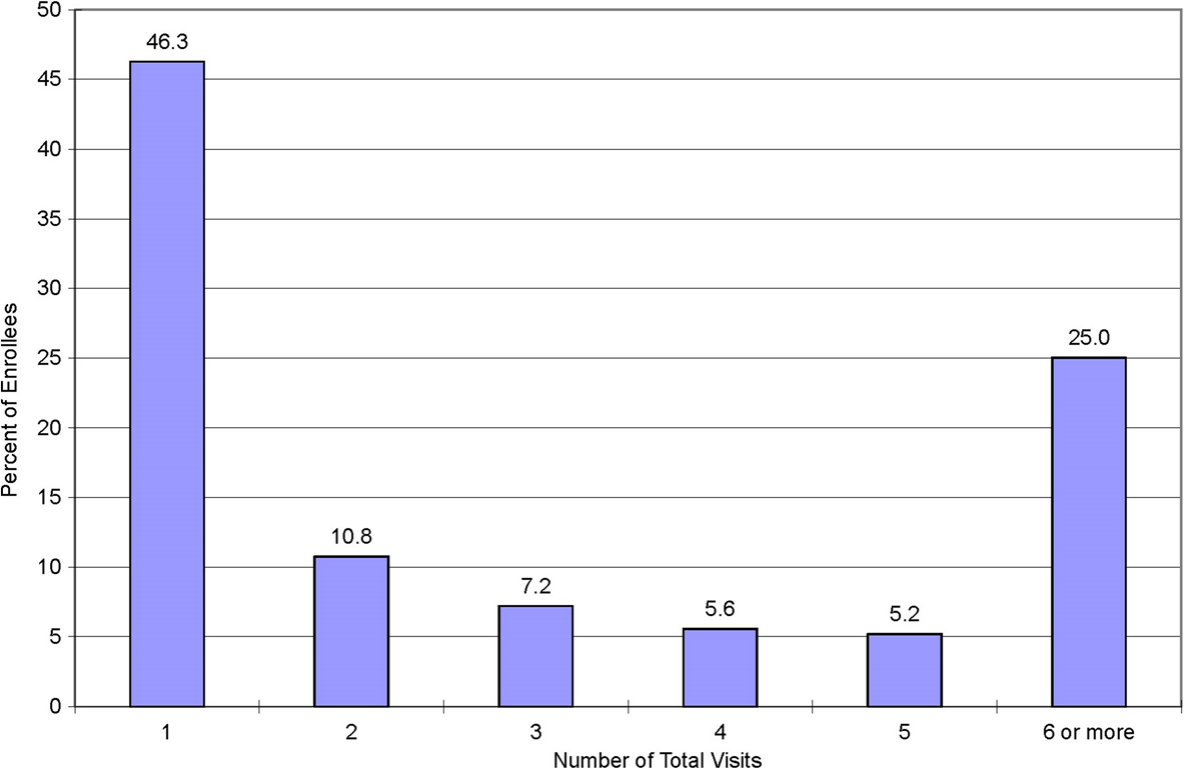
Percentage distribution of MOVE! group participants by total number of visits attended during six months after the first visit.

Forty six percent of the participants dropped out after attending just one session, 64% dropped out before the fourth visit and 74% dropped out before the sixth visit (Figure 2). Participants visited the MOVE! onsite program 4.4 times (interquartile range 1 – 6) during the six-month follow-up.

In unadjusted bivariate analyses, age, race/ethnicity, baseline BMI, and comorbidities were significantly associated with retention. While 32% of individuals ≥65 years of age completed six or more sessions, only 20% of those <55 years did (P < 0.001). Twenty-nine percent of non-Hispanic white individuals attended six or more sessions, while 23% of non-Hispanic black individuals and 18% of individuals with other or unknown race/ethnicity did (P < 0.001).

Morbidly obese individuals were more likely to complete the program than overweight individuals (29% vs 21%; P < 0.001). Whereas 27% of individuals with ≥2 comorbidities were completers, only 21% of those with no comorbidities were completers (P < 0.001). While 40% of the completers had individual consultation on or before the first group session, only 27% of the early dropouts did (P < 0.001).

Retention was greater in facilities that reported a higher staff to patient ratio (Table 2). In programs with high staffing, 31% of participants completed six or more sessions, while in those with low staffing only 18% completed six sessions (P < 0.001).

Over 75% of all sites used five or more of the ten weight management strategies we examined and a half used seven or more strategies, regardless of staffing level. Staffing was associated negatively with the number of strategies used at each site (r = −0.18; p = 0.04). While many weight management strategies were widely available, use of incentives/reward (28%), use of structured low calorie diet plans (31%), and regular onsite physical activity programming (27%), were available to less than one third of participants. The proportion of patients completing 6 or more sessions was 29% in facilities that reported using incentives or rewards, 30% in those that used low calorie diet plans, and 32% in those with onsite physical activity programming, as compared to 25% overall (all comparisons P < 0.001).

### Individual factors

Table 3 provides multivariable results for individual, facility-level, and program-level factors. The two sets of odds ratios represent the likelihood of successful short-term and long-term retention, respectively.

Compared to individuals with age < 55, those between ages 55–64 years were at least 43% more likely to stay in the program (OR_short-term_ = 1.43, 95% Confidence Interval [CI] = 1.32 – 1.55; OR_long-term_ = 1.53, 95% CI = 1.40 – 1.68). Those 75 years or older were at least 58% more likely to stay in the program (OR_short-term_ = 1.58, 95% CI = 1.39 – 1.79; OR_long-term_ = 1.83, 95% CI = 1.61 – 2.08).

Sex and BMI were uniformly associated with both short-term and long-term retention. Males were 25% more likely than females to drop out earlier (OR = 0.75, 95% CI = 0.66 – 0.84). Compared to overweight individuals, obese individuals were 22% more likely (OR = 1.22, 95% CI = 1.09 – 1.37) and morbidly obese persons were 47% more likely (OR = 1.47, 95% CI = 1.28 – 1.69) to remain in the program.

### Accessibility factors

For both short- and long-term retention, a greater geographic distance to the clinic was associated with lower retention. Compared to those living within 10 miles of the clinic, those who lived 10 or more miles away had a 25% lower likelihood of retention (OR = 0.75, 95% CI = 0.66 – 0.86). Those who lived 20–30 miles away had a 34% lower likelihood of retention (OR = 0.67, 95% CI = 0.54 – 0.82). Retention for those living more than 30 miles from the clinic was similar to those living 20–30 miles away.

### Facility factors

Offering individual consultation to group participants was uniformly associated with both short-term and long-term retention. Individual consultation was associated with increased retention regardless of when it was received. Compared to participants who did not receive it, those who received individual consultation on the same day or before starting MOVE! were 68% more likely (OR = 1.68, 95% CI = 1.30 – 2.18) and those who received it after the index date were 72% more likely (OR = 1.72, 95% CI = 1.24 – 2.38) to complete the program.

Facilities that offered self-management support through visits or by telephone had only half the retention of those without it (OR = 0.51, 95% CI = 0.34 – 0.77). Providing access to bariatric surgical services was not associated with short-term retention but was associated with 35% greater long-term retention (OR = 1.35, 95% CI = 1.01 – 1.81).

Compared to sites with low MOVE! staffing (< 3.4 full-time equivalents [FTEs] per 1,000 participants), sites with high staffing (7.61 or more FTEs) were almost twice as likely (OR = 1.96, 95% CI = 1.33 – 2.89) to retain patients in the program.

### Program strategies

Among 10 common weight loss strategies we examined, only two were associated with retention. Regular on-site physical activity program was associated with 51% greater retention (OR = 1.51, 95% CI = 1.06 – 2.16) and use of incentives/rewards was associated with 44% greater retention (OR = 1.44, 95% CI = 1.01 – 2.06).

## Discussion

Only one-fourth (26%) of all participants in the MOVE! group weight loss program attended six or more sessions during a six-month follow-up in 2008. More than 60% of all participants dropped out before the fourth session and 46% of the participants dropped out after attending just one treatment session.

Although high attrition is a common problem for weight management programs, MOVE! attrition rates appear to be higher than most. Tsai and Wadden [1] reported that attrition rates for nine weight loss programs ranged from 19% at 13 weeks to 56% at 26 weeks. All but two programs had less than 40% attrition during the initial phase that extended for 13 weeks or longer. Two programs with the highest attrition rates (45% and 56% at 26 weeks) offered on-site group counseling and 12 weeks of a very low calorie diet, respectively. Compared to these programs, an attrition rate of 74% before completing six visits represents a major challenge for achieving impact at the population level in the VA. Six of the 10 studies reviewed in Tsai and Wadden [1] were randomized controlled trials, whose initial screening and enrollment procedures may have functioned as a run-in process that screened out less motivated participants. The more selected sample in a controlled study than in a “take all comers” real world context might partially explain the much higher attrition in the MOVE! program than in other programs in the review. Nevertheless, regardless of whether the very high early attrition we observed is selective to the VA or a more general problem of real world obesity treatment programs, such attrition is clearly a barrier to optimal weight loss outcomes.

Because high attrition diminishes the effectiveness of weight management programs [1,3-6], our analysis was focused on understanding factors that affect attrition. Older age, higher number of comorbidities, and higher BMI were all associated with greater retention, whereas male sex and non-Hispanic black race were associated with lower retention.

Previous research on factors that affect attrition from weight loss treatment is limited. Honas et al. (2003) examined data from a single medical weight management program and found that female sex, divorced status, African American race, and ages <50 were associated with higher attrition [5]. Programs that promoted autonomous motivation [21] or used incentives [4] were found to increase retention, whereas high (or unrealistic) baseline weight loss expectations were found to increase attrition [2,22]. Fabricatore and colleagues (2009) reported that younger age and baseline depressive symptoms are significantly associated with increased attrition from randomized clinical trials [23].

Our results were largely consistent with previous findings, except for marital status and comorbid depression which showed no significant association with attrition in our study (data not shown). Noticeably absent from the literature is information about how variations in accessibility, program features, and provider characteristics affect attrition. This is the first study to examine facility, program, and patient characteristics conjointly as potential factors affecting attrition in medical weight management programs.

A recent study of MOVE! best practices at 22 sites [24] identified the use of a standard curriculum and care delivered wholly or partly in a group-based format as one of two necessary (but not sufficient) factors for achieving larger patient weight loss outcomes. In the presence of these two necessary conditions, four combinations of conditions were determined to be sufficient for larger patient weight loss outcomes. These combinations included a design that required both individual and group-based care as well as a high staffing level. These findings parallel our findings that individual consultation and high staffing levels were both associated with retention.

Our results have several national and local policy implications. Geographic access is often a barrier to retention when MOVE! groups are held on-site. Electronic or telephone delivery of treatment offers potential solutions. Research on the effectiveness of Internet intervention has shown mixed results [1,25-28] but obesity treatment delivery via telephone holds considerable promise [29]. TeleMOVE!, a home telehealth version of MOVE! introduced in 2010, uses a home messaging device to provide 90-day cycles of daily, automated, interactive dialog with patients in response to data they transmit. A trained care coordinator monitors progress and intercedes with the patient as needed. TeleMOVE! can be used as an alternative channel of obesity treatment delivery to overcome geographic barriers to the program. However, its effectiveness has yet to be established.

Our findings suggest that more extensive use of individual consultation with specialists is warranted as an adjunct to group treatment early in the MOVE! program. Although we still do not clearly understand the extent to which effective obesity intervention requires in-person contact (or more specifically, which patients require it), our results as well as the “best practices” evaluation study [24] highlight the value of in-person contact and supplementing group sessions with individual consultation for both retention and outcomes.

Of the program factors we examined, regular on-site physical activity was the only strategy associated with increased retention. Currently, many sites do not have adequate resources for providing regular physical activity. According to the NCP Annual Report, 67% of facilities reported that indoor physical activities were barely or not at all sufficient for MOVE! program needs and 37% reported that outdoor physical activity facilities were insufficient.

Based on these results, we suggest that the VHA consider adopting a measure of MOVE! treatment retention as a facility performance or quality indicator. Current MOVE!-related measures focus on screening for obesity and participation in at least one treatment visit, but they do not recognize or reward facilities for better retention. In 2011, the VHA introduced retention as a pilot indicator of site performance. Future research should examine the effect of this indicator on attrition and, ultimately, on patient weight loss.

Major strengths of our study were its large sample and simultaneous modeling of individual, facility, and program characteristics associated with retention in an evidence-based group obesity treatment program. A limitation is that data for facility and program characteristics were drawn from the NCP Annual Report that is primarily used to monitor accountability within the healthcare system. This required report is completed by each facility’s MOVE! coordinator, and the fidelity with which facilities implemented each program strategy has not been validated. Also, because our study design was observational, causal inferences cannot be drawn.

## Conclusion

We found a number of individual, facility, and program characteristics to be associated with retention in the MOVE! group treatment program. Individual attributes such as older age, more comorbidities, and higher BMI were associated with greater retention, whereas male sex, non-Hispanic black race, and living a greater distance from the MOVE! facility were associated with lower retention. Facility factors including offering of individual consultation and more MOVE! staff per participant were associated with higher retention. Among the program characteristics we examined, holding a regular on-site physical activity sessions and offering incentives/rewards were associated with higher retention.

Recognizing that certain characteristics place patients at heightened risk for drop-out may help in identifying those who need targeted outreach to retain them in treatment. Optimal targeted retention strategies for at risk demographic groups still need to be developed, as do those more personally tailored for individuals. As an example of targeting, offering TeleMOVE! or telephone treatment to those who live at a greater distance from the treatment facility might prove warranted. The findings also suggest that to the extent possible given a facility’s resource constraints, optimizing facility level factors so as to offer individual consultation and a higher provider to patient staffing ratio may help foster retention. At the level of staff decisions about obesity program design, investment in onsite exercise programming and incorporation of incentives into program design warrant additional study as ways to improve retention.

Although greater attention to these factors may help improve treatment retention and weight loss outcomes, a non-completion rate of 74% for in-person group obesity treatment poses a major challenge to reducing the population prevalence of obesity within the VHA. In the future, supplementing the VA’s in-person group treatment with connective technology offers hope for improved weight loss [30]. Purely technology-based programs such as TeleMOVE! may also be well-utilized to provide weight loss interventions at home for patients whose access to on-site treatment is limited. Given that attrition currently hinders the effectiveness of the MOVE! onsite program and that long-term retention is low, more research is needed to find new ways of improving retention in the MOVE! onsite program. Effective use of reminders for patients about upcoming MOVE! sessions, combined implementation of MOVE! groups plus TeleMOVE! or mobile health tools warrant investigation as ways to help to improve patients’ engagement in weight loss programming.

## Abbreviations

VHA: Veterans Health Administration; USPSTF: United States Preventive Services Task Force; NIH: National Institutes of Health; ADUSH: Assistant Deputy Under Secretary of Health; NCP: VA National Center for Health Promotion and Disease Prevention; BMI: Body Mass Index; CDW: Corporate Data Warehouse.

## Competing interests

The authors declare that they have no competing interests.

## Authors’ contributions

BS, MS, SL, SH, LK and FW participated in the conception and design of the study, critical review of the manuscript, and interpretation of results; MS acquired the data and conducted statistical analysis; MS and SL drafted the manuscript; All authors approved the manuscript.

## Pre-publication history

The pre-publication history for this paper can be accessed here:

http://www.biomedcentral.com/1471-2458/14/363/prepub
